# Anti-inflammatory effect of lavender (*Lavandula angustifolia* Mill.) essential oil prepared during different plant phenophases on THP-1 macrophages

**DOI:** 10.1186/s12906-021-03461-5

**Published:** 2021-11-24

**Authors:** Edina Pandur, Alex Balatinácz, Giuseppe Micalizzi, Luigi Mondello, Adrienn Horváth, Katalin Sipos, Györgyi Horváth

**Affiliations:** 1 Department of Pharmaceutical Biology, Faculty of Pharmacy, University of Pécs, H-7624, Rókus u. 2, Pécs, Hungary; 2 Department of Pharmacognosy, Faculty of Pharmacy, University of Pécs, H-7624, Rókus u. 2, Pécs, Hungary; 3 Chromaleont s.r.l., c/o Department of Chemical, Biological, Pharmaceutical and Environmental Sciences, University of Messina, 98168 Messina, Italy; 4 Department of Chemical, Biological, Pharmaceutical and Environmental Sciences, University of Messina, 98168 Messina, Italy; 5 Unit of Food Science and Nutrition, Department of Medicine, University Campus Bio-Medico of Rome, 00128 Rome, Italy

**Keywords:** Lavender, Essential oil, Inflammation, Macrophage, Cytokines, NFκB inhibitor

## Abstract

**Background:**

*Pseudomonas aeruginosa* is the most common Gram-negative bacterium associated with nosocomial respiratory infections. Lavender essential oil is mainly used in aromatherapy, but it has several pharmacological and therapeutic properties. Furthermore, it possesses antifungal and antibacterial activities. The anti-inflammatory activity of essential oils may depend on the composition and the ratio of the compounds. The constitution of the essential oils extracted from the different stages of flowering period varies, which makes it plausible that the collection time of the flowers influences the anti-inflammatory effects. Different types of essential oils reduce inflammation acting similarly by modulating the activity and action of the NFκB signalling pathway, which is the major regulator of the transcription of pro-inflammatory cytokines.

**Methods:**

Lavender essential oils were distilled from lavender plant cultivated in Hungary and the flowers were harvested at the beginning and at the end of flowering period. The experiments were carried out on THP-1 human monocyte/macrophage cell line as in vitro cell culture model for monitoring the effects of lavender essential oils and the main compound linalool on *P. aeruginosa* LPS stimulated inflammation. The mRNA and protein levels of four pro-inflammatory cytokines, IL-6, IL-1β, IL-8 and TNFα were determined by Real Time PCR and ELISA measurements. The effects of essential oils were compared to the response to two NFκB inhibitors, luteolin and ACHP.

**Results:**

Linalool and lavender essential oil extracted from plants at the beginning of flowering period were successful in decreasing pro-inflammatory cytokine production following LPS pretreatment. In case of IL-8 and IL-1β lavender oil showed stronger effect compared to linalool and both of them acted similarly to NFκB inhibitors. Pretreatments with linalool and lavender essential oil/beginning of flowering period prevented pro-inflammatory cytokine production compared to LPS treatment alone. Although lavender essential oil/end of flowering period decreased IL-6, IL-1β and IL-8 mRNA expression in case of LPS pretreatment, it was not capable to reduce cytokine secretion.

**Conclusion:**

Based on our results it has been proven that lavender essential oil extracted at the beginning of flowering period is a potent inhibitor of the synthesis of four pro-inflammatory cytokines IL-6, IL-8, IL-β and TNFα of THP-1 cells. This supports the relevance of the collection of the lavender flowers from early blooming period for essential oil production and for the utilization as an anti-inflammatory treatment.

**Supplementary Information:**

The online version contains supplementary material available at 10.1186/s12906-021-03461-5.

## Background

*Pseudomonas aeruginosa* is the most common Gram-negative bacterium associated with nosocomial respiratory infections [1] and it is frequently associated with cystic fibrosis and chronic inflammatory airway diseases [2–4]. *P. aeruginosa* strains have intrinsic and acquired antibiotic resistance due to the capability of biofilm and multidrug-tolerant cell formation [5, 6]. Although nebulised antibiotics seem to be effectual at early *P. aeruginosa* infection alternative therapeutic strategies are highly required [7].

Monocytes are part of the innate immune system. Upon activation they may differentiate towards M1 type with pro-inflammatory phenotype and phagocytic activity or M2 type promoting cell proliferation and tissue repair [8]. Lipopolysaccharide (LPS) found in the cell wall of various bacterium strains activates M1 polarization of the monocytes, which begin to release pro-inflammatory cytokines such as interleukin-6 (IL-6), interleukin-1β (IL-1β), interleukin-8 (IL-8) and tumor necrosis factor α (TNFα) as well as prostaglandins [9]. LPS binds to Toll-like receptor 4 (TLR4) and activates MyoD88 dependent and independent signalling pathways. The former activates nuclear factor kappaB (NFκB) transcription factor and mitogen activated protein kinase/activator protein-1 (MAPK/AP-1) signalling, the latter regulates interferon regulatory factor 3 (IRF3), but both of them trigger pro-inflammatory cytokine transcription [10].

According to literature, the essential oils like thyme, chamomile and eucalyptus reduce inflammation by modulating the activity and action of the NFκB signalling pathway, which is the major regulator of the transcription of pro-inflammatory cytokines [11–13].

Several essential oils e.g. tea tree oil, eucalyptus oil and lavender oil possess immunomodulatory activity, which can alter the inflammatory processes that makes them a potential alternative treatment of many infectious or immune diseases [14, 15]. Immunosuppression by essential oils may contribute to their anti-tumour activities [16]. Inhalation of certain essential oils can attenuate respiratory tract inflammation [17, 18].

The essential oils are usually extracted from the main flowering period of many plants because these flowers usually provide the highest essential oil yield and it is hypothesised that these essential oils comprise the most effective mixture of the different compounds [19–22]. In the contrary, in some years due to weather anomalies or due to the global warming, it would be favourable to collect the flowers at the beginning as well as at the end of flowering period at certain cultivation area. The production of essential oil at these time points of flowering period would be beneficial at economic aspect as well if these essential oils show better or similar activities in the field of utilization compared to those, which are produced at the main flowering period. According to ESCOP Monograph and European Medicines Agency (EMA) lavender essential oil (LEO) is obtained by steam distillation from the fresh flowers of *Lavandula angustifolia* Mill [23, 24].. The therapeutic indications of lavender oil include the relief of mild symptoms of mental stress and exhaustion and the treatment of insomnia. Therefore, today the LEO and its products are among the most popular oils worldwide. Linalool is the major natural compound of lavender oil. Recent studies have described its antibacterial [25] and anticancer effect [26]. However, data about the mechanism of anti-inflammatory activity of linalool and lavender oil is limited on human cell lines, e.g. THP-1.

The experiments were carried out on THP-1 human monocyte/macrophage cell line that is widely used as in vitro cell culture model for monitoring the effects of plant extracts on LPS stimulated inflammation [27–29].

Our study revealed that essential oils distilled from lavender flowers collected at the beginning and at the end of the flowering period could influence the pro-inflammatory cytokine production of *P. aeruginosa* LPS activated THP-1 cells. The essential oils used in the experiments were distilled from fresh lavender plant cultivated in Hungary (Bolho village, Somogy County) and the flowers were harvested at the beginning and at the end of flowering period. Lavender was the medicinal plant of the year in 2018, in Hungary [30]. However, until today nobody evaluated scientifically the lavender growing on this cultivation field.

To prove the effectiveness of linalool, the main compound of LEO, and the LEOs prepared at the beginning and at the end of flowering period and to reveal whether these oils can attenuate pro-inflammatory cytokine expression via the NFκB pathway in THP-1 cells, two specific NFκB inhibitors luteolin and ACHP were used. These inhibitors block NFκB pathway at different points [31–34].

Based on our results it seems that the differences in the composition of lavender oil extracted at beginning and at the end of flowering period may participate in their distinct effects on the gene expression regulation and secretion of the four examined pro-inflammatory cytokines in *P. aeruginosa* LPS-stimulated THP-1 cells. It has been proven that LEO distilled from the flowers and harvested at the beginning of flowering period is a potent inhibitor of the synthesis of IL-6, IL-8, IL-β and TNFα of THP-1 cells, which makes it a good candidate as an alternative anti-inflammatory therapy.

## Methods

### Plant material and essential oil distillation

*Lavandula angustifolia* Mill. was collected in two phenophases of the plant: at the beginning of the flowering period (20 June, 2019) and at the end of the flowering period 18 July, 2019). The location of the plant harvesting was: Bolho village (Somogy county, Hungary, coordinates: 46.03904°N 17.30376°E). LEO was obtained from the fresh (not dried) plant material by hydrodistillation according to the Hungarian Pharmacopoeia 8th edition (2003) in 2019. The duration of the procedure was 3 h. The LEO content was measured with the volumetric method. Three distillation procedures were prepared parallel. From the fresh plant material collected at the beginning of flowering period 1810 μL LEO and from the fresh plant material collected at the end of flowering period 1100 μL LEO was obtained, respectively. The chemical composition of the LEO samples was determined by gas chromatography coupled to single-quadrupole mass spectrometer (GC-MS) and flame ionisation detector (GC-FID).

### LEO samples for GC-MS and GC-FID

LEO samples (10 μL) were solubilized in 990 μL methanol (Sigma-Aldrich Kft., Budapest, Hungary) (dil. 1:100) and injected into the GC-MS and GC-FID systems. A C7-C30 saturated alkanes (1000 μg/mL) standard mixture in hexane (Sigma-Aldrich Kft., Budapest, Hungary) was utilized for linear retention index (LRI) calculation.

### GC-MS

The separation and identification of the LEO samples was carried out by using a GCMS-QP2020 instrument (Shimadzu, Duisburg, Germany) equipped with a split-splitless injector (280 °C) and an AOC-20i auto-sampler. The capillary column was a low-polarity one, namely SLB-5 ms 30 m × 0.25 mm id, 0.25 μm df (Sigma-Aldrich Kft., Budapest, Hungary). The temperature program was as follows: 50 °C to 300 °C at 3.0 °C/min. Injection volume was 0.5 μL with a split ratio of 1:10. Helium was used as carrier gas, at an initial inlet pressure of 26.7 kPa and at an average linear velocity of 30 cm/s. The MS parameters were as follows: the mass range was 40–550 amu, the ion source temperature was 220 °C, and the interface temperature was 250 °C. The GCMSsolution software (version 4.50 Shimadzu) was used for data collection and handling. Peak assignment was carried out through the application of a double filter: spectral similarity (over 85%) and a ± 5 LRI tolerance window. The FFNSC mass spectral library version 3.01. (Shimadzu, Europe, Duisburg, Germany) was mainly used for the compound identification [35].

### GC-FID

The quantification of analytes was carried by using a GC-2010 instrument (Shimadzu, Europe, Duisburg, Germany) equipped with a split-splitless injector, an AOC-20i/s auto-sampler and an FID detector (280 °C). The GC column, temperature program, and carrier gas were the same as described for the GC-MS system, apart from the initial inlet pressure (99.5 kPa) (the average linear velocity was 30 cm/s). The FID temperature was set at 300 °C (sampling rate: 40 ms), while the gas flows were 40 mL/min for H_2_, 30 mL/min for the make-up gas (N_2_) and 400 mL/ min for air. Data were collected and processed using the LabSolution software (version 5.92, Shimadzu). Each sample was analysed for three consecutive runs for a major precision of data [35].

### LEO samples for cell line studies

Stock solutions of linalool standard (Sigma-Aldrich Kft., Budapest, Hungary) and LEOs were prepared by adding 10% of 100% dimethyl sulphoxide (DMSO, Sigma-Aldrich Kft., Budapest, Hungary) to 90% of essential oils, which means that 100 μL of DMSO was added to 900 μL of essential oil in a final volume of 1 mL. The emulsions were mixed by vortexing then were diluted with phosphate buffered saline (PBS, Lonza Ltd., Basel, Switzerland) 500-fold, 1000-fold, 2000-fold and 3000-fold. Stock solutions and the dilutions were prepared freshly in each experiment. For control experiments stock solution containing 10% of DMSO was prepared in PBS and was diluted the same way as the essential oils. The final concentration of DMSO used in the experiments was equal to 0.01% or less according to the dilutions.

### Cell culture and treatments

THP-1 human monocyte/macrophage cell line was purchased from the European Collection Authenticated Cell Cultures (Sigma-Aldrich Kft., Budapest, Hungary). The cells were cultured in RPMI-1640 medium supplemented with 10% fetal bovine serum (FBS; EuroClone S.p.A, Pero, Italy) and 1% Penicillin/Streptomycin (P/S; Lonza Ltd., Basel, Switzerland) in a humidified atmosphere containing 5% CO_2_ at 37 °C. THP-1 cells were placed into 6-well plates and were cultured for 24 h before the treatments. The inflammation was generated by treatments using 100 ng/mL *Pseudomonas aeruginosa* LPS (*Pseudomonas aeruginosa* 10 purified by phenol extraction; Sigma-Aldrich Kft., Budapest, Hungary). The concentration of *P. aeruginosa* LPS and the duration of the experiments were determined by concentration and time dependence experiments (Supplementary Fig. 1.). The concentrations of the NFκB inhibitors luteolin (Tocris Bioscience, Bio-Techne R&D Systems Kft., Budapest, Hungary) and ACHP (2-Amino-6-[2-(cyclopropylmethoxy)-6-hydroxyphenyl]-4-(4-piperidinyl)-3-pyridinecarbonitrile; Tocris Bioscience, Bio-Techne R&D Systems Kft., Budapest, Hungary) used as control of the inhibition of cytokine production was determined by concentration dependence experiments (Supplementary Fig. 2.). The cells were treated with 500-fold diluted linalool standard and LEOs to determine their effects on pro-inflammatory cytokine production. First the effect of linalool standard and LEOs on pro-inflammatory cytokine expression was determined on the THP-1 cell without LPS pretreatment. The anti-inflammatory effects of essential oils were determined in two different experiments: LPS pretreatment for 24 h then essential oil treatment for 24 h; essential oil pretreatment for 24 h then LPS treatment for 24 h. DMSO treated cells were used as controls. The final concentration of DMSO was 0.01% according to the dilution in the experiments. Each experiment was repeated three times.

### Cell viability measurements

Viability of the THP-1 cells were measured using Cell Counting Kit-8 (CCK-8) cell viability assay (Sigma-Aldrich Kft., Budapest, Hungary) after the treatments. The cells were seeded into 96-well plates using 5 × 10^3^ cells/well. Cells were treated with either linalool or LEOs in 500-fold, 1000-fold, 2000-fold or 3000-fold dilutions for 6 h and 24 h. The dilutions were made freshly form the stock solutions described earlier in the methods section. DMSO treated cells were used as controls of the essential oil treated cells. The final concentrations of DMSO used in the experiments were equal or less than 0.01% according to the dilutions. In the assay, according to the protocol, 10 μL of WST-8 reagent was added to each well containing the treated cells then the plates were incubated for 1 h at 37 °C and 5% CO_2_. After incubation, the reaction was stopped using 10 μL of 1% sodium-dodecyl sulphate (SDS, Molar Chemicals Kft., Halásztelek, Hungary). The absorbance of the samples was measured at 450 nm using MultiSkan GO microplate spectrophotometer (Thermo Fisher Scientific Inc., Waltham, MA). Viability of the treated cells was expressed as percentile of the total cell number of the appropriate DMSO control [36].

### Real time PCR analysis

THP-1 cells were treated with 500-fold diluted linalool or LEOs in 6-well culture dishes using 4 × 10^5^ cells/well as described earlier. After the treatments, THP-1 cells were harvested by centrifugation, the supernatants were collected in new tubes for ELISA measurements. The cell pellets were washed twice with PBS then total RNA was isolated from each sample using Quick RNA mini kit (Zymo Research, Irvine, CA). The cDNA was synthesised from 200 ng of total RNA using High capacity cDNA Reverse Transcription Kit in a 20 μL of total reaction volume (Applied Biosystems, Thermo Fisher Scientific Inc., Waltham, MA) according to the manufacturer’s protocol. Determination of gene expressions was performed in a CFX96 Real-time System (Bio-Rad Inc., Hercules, CA) using iTaq™ Universal SYBR® Green Supermix (Bio-Rad Inc., Hercules, CA). Each reaction contained 2 μL (20 ng) of cDNA, 10 μL of 2X Master mix, 10 μM forward and reverse primers and 7.2 μL of ultrapure water. To prove specificity of the PCR reactions, melting curves were generated after each run. Relative quantification was calculated by the Livak (2^-∆∆Ct^) method using the Bio-Rad CFX Maestro 1.1. software (Bio-Rad Inc., Hercules, CA) in which the mRNA level of β-actin house-keeping gene was used for normalization. The relative expression of the controls (DMSO treated cells) was regarded as 1. The mRNA expression of the treated cells were compared to the controls and was expressed as fold change [36]. The primer sequences used in this study are described in Table 1.

**Table 1:** Real-time PCR gene primers used in the experiments

Primer	Sequence 5′ → 3’
IL-6 forward	CTGAGAAAGGAGACATGTAACAAG
IL-6 reverse	GGCAAGTCTCCTCATTGAATC
IL-8 forward	CAGTGCATAAAGACATACTCC
IL-8 reverse	CACTCTCAATCACTCTCAGT
IL-1β forward	GAAATGATGGCTTATTACAGTGG
IL-1β reverse	GGTGGTCGGAGATTCGTA
TNFα forward	CTCTCTCTAATCAGCCCTCT
TNFα reverse	CTTGAGGGTTTGCTACAACA
β-actin forward	AGAAAATCTGGCACCACACC
β-actin reverse	GGGGTGTTGAAGGTGTCAAA

### Enzyme-linked immunosorbent assay (ELISA) measurements

The cells were pelleted after each treatment and the supernatant of the cells were placed into new tubes. The culture medium samples were stored at −80 °C until the ELISA measurements. The concentrations of IL-6, IL-1β, IL-8 and TNFα were determined in triplicate in each independent experiment using human IL-6, IL-1β, IL-8 and TNFα specific ELISA kits (Thermo Fisher Scientific Inc., Waltham, MA) according to the instructions of the manufacturer.

### Statistical analysis

The cell viability assays, the Real-time PCR analyses and ELISA measurements were carried out in triplicate in each independent experiments. In each figure, *n* corresponds to the number of the independent experiments. Statistical analysis was performed using SPSS software (IBM Corporation, Armonk, NY, USA). Statistical significance was determined by Kruskal-Wallis one-way ANOVA non-parametric test using pairwise comparisons. Data are shown as mean ± standard deviation (SD). Statistical significance was set at *p* value < 0.05*.*

## Results

### Effects of linalool and LEOs on cell viability of THP-1 cells

**Fig. 1:**
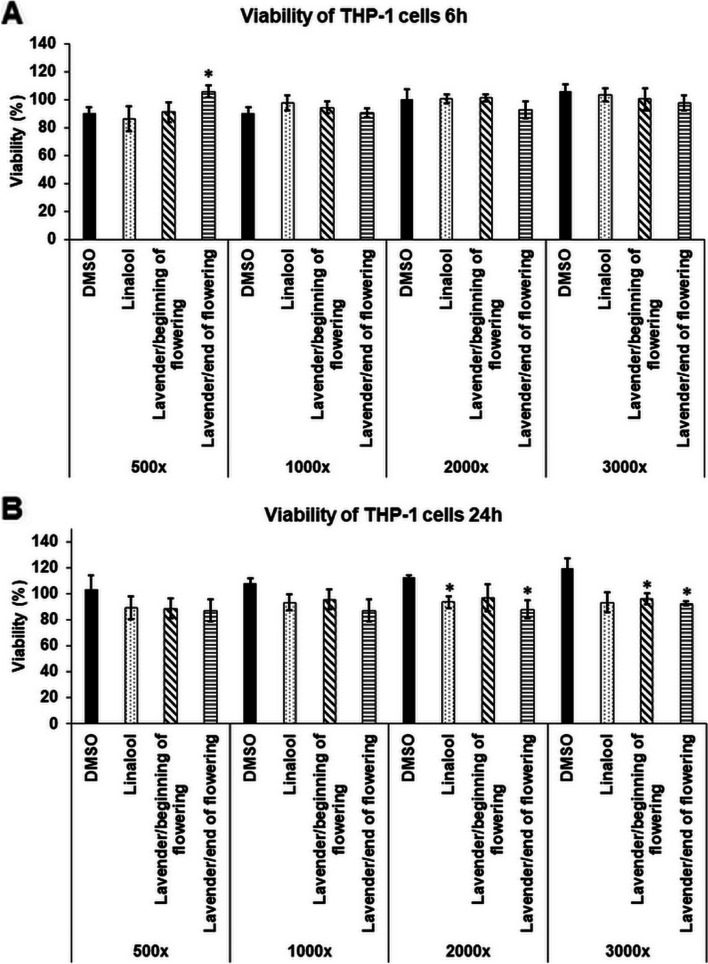
Cell viability determinations of THP-1 cells treated with linalool and LEOs. Viability of the THP-1 cells was measured using CCK-8 cell viability assay after 6 h (**A**) and 24 h (**B**) long treatments with DMSO, linalool and LEOs using serial dilutions of the stock solutions. Viability is expressed as percentile of the total cell number of the control cells. The bars represent mean values and error bars represent standard deviation (SD) for four independent experiments (*n* = 4). Cell viability assays were carried out in triplicate in each experiment. Asterisks indicate *p* < 0.05 compared to the DMSO treated cells used as controls. The diagrams were created using the same logical structure and the different treatments appeared in the same sequence in each figure in order to improve traceability of the results

To examine the anti-inflammatory effects of linalool standard and LEOs on LPS-induced THP-1 cells, the cytotoxic effects of the essential oils were determined. The cytotoxicity was defined by the measurement of the viability of the cells after 6 h and 24 h long treatments using serial dilutions of the stock solutions prepared from linalool and LEOs (Fig. 1A,B). DMSO was used for emulsification, therefore the DMSO treated cells were used as controls. After 6 h long treatments, significant decrease of cell viability was not measured (Fig. 1A). The longer treatments only affected cell viability at higher dilutions compared to the same dilution level of DMSO, which may be due to the proliferation activating effect of DMSO (Fig. 1B). Based on these results the lowest dilution rate (500-fold) of linalool and LEOs, which did not affect cell viability significantly, was chosen for the further experiments. The cell viability may depend on the composition of the essential oils and the concentration of the compounds (Table 2).

**Table 2:** Percentage (%) and relative concentrations (ng/mL) of the compounds of LEOs prepared at the beginning and at the end of the flowering period

		Percentage of compound in the lavender essential oils^a^		Relative concentration of compound in the experiments (ng/mL)^b^	
Compounds	LRI_exp_	before flowering period	end of flowering period	before flowering period	end of flowering period
Tricyclene	923	0.02	0.01	0.34	0.17
α-Thujene	925	0.18	0.2	3.36	3.74
α-Pinene	933	0.36	0.32	6.33	5.62
Camphene	949	0.34	0.19	6.12	3.42
Sabinene	972	0.11	0.06	1.98	1.08
β-Pinene	977	0.15	0.06	6.54	2.62
Octan-3-one	984	0.35	0.75	5.60	12.00
Myrcene	988	1.62	1.35	25.90	21.60
Butyl-butanoate	994	0.04	0.02	0.72	0.36
*n*-Decane	999	0.03	0.03	0.42	0.42
α-Phellandrene	1006	0.02	0.02	0.34	0.34
δ-3-Carene	1009	0.27	0.09	4.67	1.56
Hexyl-acetate	1011	0.15	0.04	2.61	0.69
α-Terpinene	1017	0.03	0.02	0.48	0.32
*o*-Cymene	1019	0.05	0.03	0.88	0.53
*p*-Cymene	1024	0.67	0.81	11.52	13.93
Limonene	1029	1.1	0.62	18.52	10.44
β-Phellandrene	1030	0.24	0.06	3.93	0.98
Eucalyptol	1032	0.48	0.32	8.84	5.89
(Z)-, β-Ocimene	1034	3.43	1.33	53.23	20.64
(E)-, β-Ocimene	1045	0.77	0.48	12.32	7.68
γ-Terpinene	1058	0.02	0.02	0.32	0.32
(Z)-Linalool oxide	1070	0.18	0.16	3.40	3.02
Terpinolene *	1086	0.04	0.26	0.72	4.68
(E)-Linalool oxide *	1087				
*p*-Cymenene *	1091	0.11	0.11	1.92	1.92
α-Naginatene *	1091				
Linalool	1099	36.33	41.63	632.00	724.00
3-Acetoxy-octene	1107	0.8	0.76	14.40	13.68
*allo*-Ocim-(4E,6Z)-ene	1128	0.58	0.19	9.41	3.08
(E)-Myroxide	1139	0.09	0.06	1.58	1.06
Hexyl-isobutyrate	1146	0.03	0.03	0.52	0.52
Camphor	1148	0.29	0.36	5.80	7.20
Nerol oxide	1152	0.03	0.03	0.57	0.57
Terpinen-4-ol	1182	9.47	16.69	176.71	311.43
Cryptone	1188	0.87	0.27	16.20	5.03
Hexyl-butyrate	1190	0.59	0.62	10.62	11.16
α-Terpineol	1197	4.27	5.23	80.28	98.32
Bornyl formate	1230	0.02	0.03	0.41	0.61
Neral	1239	0.01	0.02	0.17	0.34
Cuminaldehyde	1244	0.11	0.05	2.20	1.00
Carvone	1245	0.11	0.06	1.98	1.08
Linalyl acetate	1249	21.47	14.78	386.46	266.04
Geraniol	1252	0.11	0.19	1.98	3.42
Geranial	1269	0.05	0.03	0.86	0.51
Lavandulyl acetate	1283	2.35	1.1	44.51	20.83
Bornyl acetate	1285	0.18	0.14	3.60	2.80
Hexyl-tiglate	1325	0.06	0.07	1.07	1.25
α-Terpinyl acetate	1347	0.07	0.07	1.26	1.26
(Z)-Geranyl acetate	1357	1.64	1.46	29.52	26.28
(E)-Geranyl acetate	1377	3.19	2.92	57.42	52.56
Hexyl-hexanoate	1385	0.01	0.03	0.18	0.54
7-*epi*-Sesquithujene	1387	0.03	0.03	0.54	0.54
α-(Z)-Bergamotene	1413	0.03	0.01	0.53	0.18
α-Santalene *	1419	1.98	1.18	35.64	21.24
(E)-Caryophyllene *	1421				
α-, (E)-Bergamotene	1433	0.08	0.04	1.42	0.71
Coumarin	1439	0.02	0.01	0.48	0.24
α-Himachalene	1444	0.04	0.03	0.80	0.60
*epi*-, β-Santalene	1447	0.04	0.02	0.71	0.36
(E)-, β-Farnesene	1452	1.51	1.3	24.37	20.98
α-Humulene	1457	0.07	0.04	1.19	0.68
Sesquisabinene	1458	0.04	0.01	0.72	0.18
(Z)-Muurola-4(14),5-diene	1463	0.03	0.01	0.54	0.18
Germacrene D	1483	0.05	0.03	0.85	0.51
β-, (E)-Bergamotene	1484	0.04	0.03	0.71	0.53
γ-Cadinene	1514	0.65	0.33	11.70	5.94
(E)-Calamenene	1522	0.06	0.03	1.20	0.60
Caryophyllene oxide	1585	0.9	0.98	18.00	19.60
1-,10-di-*epi*-Cubenol	1618	0.05	0.06	0.95	1.14
1,2,3,4,4a,7,8,8a-Octahydro-, 4-isopropyl-, 1,6-dimethyl-naphth-1-ol	1645	0.31	0.79	6.66	16.97
**Total**		99.35	99.27		

^a^LRI_exp_ – experimental linear retention index on SLB-5MS column

The volatile compounds are expressed in % values (average of three replicated injections).

^b^The relative concentrations were chosen based on the calculation as if the cells were treated with 200 μL essential oil

* indicates a coelution on SLB-5 ms column

### Effects of linalool and LEOs on mRNA expression and secretion of pro-inflammatory cytokines IL-6, IL-8, IL-1β and TNFα

**Fig. 2:**
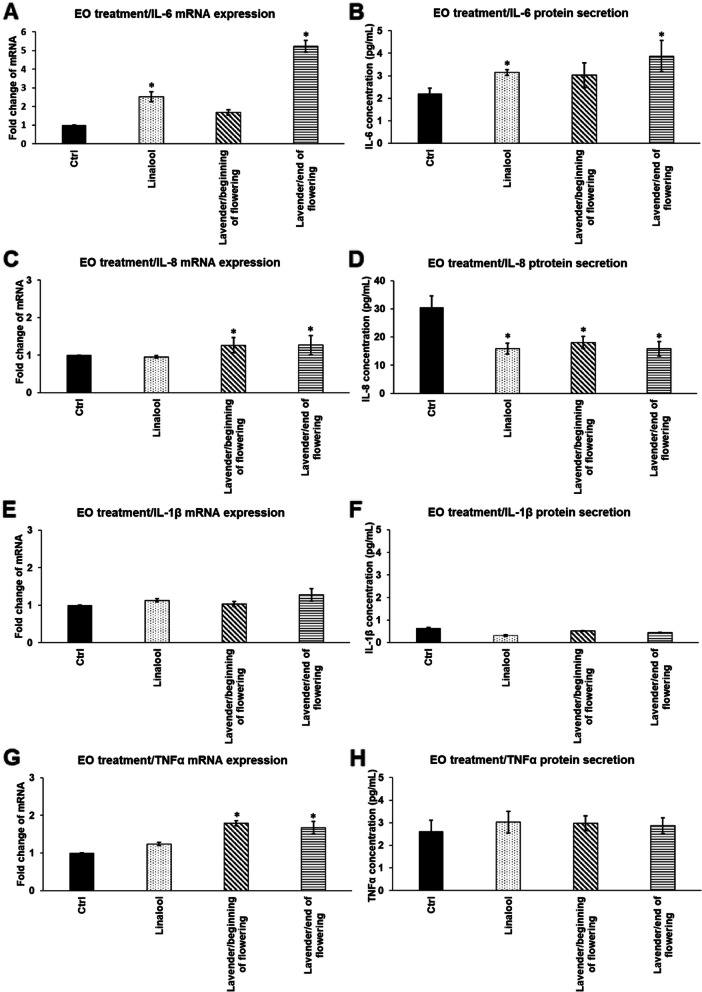
mRNA expression (**A,C,E,G**) and protein (**B,D,F,H**) levels of pro-inflammatory cytokines IL-6, IL-8, IL-1β and TNFα after linalool and LEOs treatments of THP-1 cells. THP-1 cells were treated with 500-fold diluted linalool and LEOs prepared at the beginning and at the end of flowering period. DMSO treated cells were used as a control of the essential oil treated cells. Real Time PCR was performed with SYBR green protocol using pro-inflammatory cytokine specific primers. β-actin was used as housekeeping gene for the normalization. Relative expression of controls was considered as 1. Pro-inflammatory cytokine secretions were determined using IL-6, IL-8, IL-1β and TNFα specific ELISA kits according to the manufacturer’s instructions. The bars represent mean values and error bars represent standard deviation (SD) for three independent determinations (*n* = 3). Real Time PCR and ELISA measurements were carried out in triplicate in each independent experiment. Asterisks indicate *p* < 0.05 compared to the control. The diagrams were created using the same logical structure and the different treatments appeared in the same sequence in each figure in order to improve traceability of the results

First, the effects of linalool and LEOs were determined on mRNA levels of the pro-inflammatory cytokines, IL-6, IL-8, IL-1β and TNFα in THP-1 cells. Linalool did not changed significantly the mRNA expression of cytokines except IL-6, which increased after 24 h treatment (Fig. 2A). LEO that was prepared at the beginning of flowering period increased significantly both IL-8 and TNFα mRNA expressions (Fig. 2C,G), while LEO prepared at the end of the flowering period increased all of the examined pro-inflammatory cytokine mRNA expression, except IL-1β (Fig. 2A,C,E,G). These results suggest that the composition of LEOs may have influence on the transcription of the different pro-inflammatory cytokines.

The secreted cytokine levels were also measured to reveal whether the different oils provide different actions on the protein synthesis of the pro-inflammatory cytokines. The amount of the secreted proteins followed the mRNA expression levels of cytokines in general. The IL-6 level was elevated after the treatment with linalool and LEO/end of flowering period (Fig. 2B), meanwhile both essential oils and linalool decreased IL-8 levels (Fig. 2D). The examined essential oils did not change the IL-1β secretion or TNFα production (Fig. 2F,H). These results show that LEO that was distilled at the beginning of flowering is suitable as treatment of THP-1 cells, since it did not increase the expression and production of the examined pro-inflammatory cytokines suggesting that it did not work as an activator of the monocytes. Moreover it was able to decrease the IL-8 synthesis of the cells.

Inhibitory effect of linalool, LEOs and NFκB inhibitors luteolin and ACHP on mRNA expression and secretion of pro-inflammatory cytokines after *P. aeruginosa* lipopolysaccharide pretreatment.

Next, we examined the attenuating effects of linalool and LEOs on the pro-inflammatory cytokine production after the activation of the THP-1 cells using *P. aeruginosa* LPS for 24 h. To reveal the effectiveness of the essential oils, luteolin and ACHP, two NFκB inhibitors with different mechanism of actions, were used as positive control of the downregulation of the cytokine expression.

**Fig. 3:**
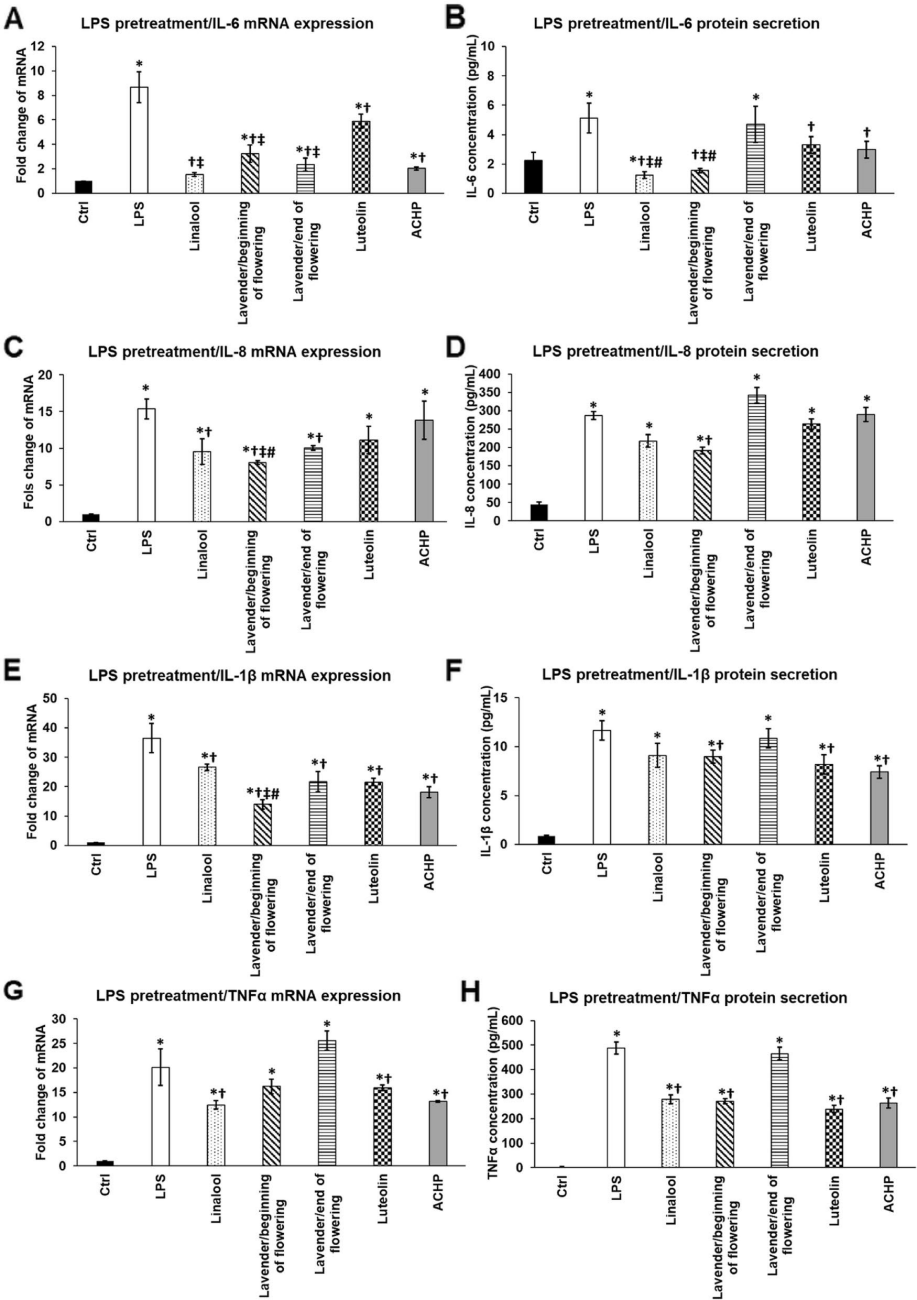
Effects of linalool, LEOs prepared at the beginning and at the end of the flowering period and NFκB inhibitors on mRNA (**A,C,E,G**) and protein (**B,D,F,H**) levels of pro-inflammatory cytokines IL-6, IL-8, IL-1β and TNFα after *P. aeruginosa* LPS pretreatment. THP-1 cells were pretreated with 100 ng/mL LPS for 24 h then 500-fold diluted linalool and LEOs or 5 μM luteolin or ACHP were added to the pretreated THP-1 cells for an additional 24 h. DMSO treated cells were used as control of the treated cells. Real Time PCR was carried out with SYBR green protocol using pro-inflammatory cytokine specific primers. β-actin was used as housekeeping gene for the normalization and relative expression of controls was regarded as 1. Pro-inflammatory cytokine secretions were determined using specific ELISA kits according to the manufacturer’s protocols. The columns represent mean values and error bars represent standard deviation (SD) for three independent determinations (*n* = 3). Real Time PCR and ELISA measurements were carried out in triplicate in each independent experiment. Asterisks indicate *p* < 0.05 compared to the control. Crosses show *p* < 0.05 compared to the LPS treatment. Double cross represent statistical significance (*p* < 0.05) compared to luteolin treatment. Number sign marks *p* < 0.05 compared to ACHP treatment. The diagrams were created using the same logical structure and the different treatments appeared in the same sequence in each figure in order to improve traceability of the results

Both examined essential oils were successful in decreasing the mRNA level of IL-6 compared to LPS treatment (Fig. 3A). Moreover, they provided a better effect than luteolin and were almost as efficient as ACHP suggesting that these essential oils act similarly on the NFκB pathway as ACHP. At protein level, linalool and LEO/beginning of flowering were more auspicious in decreasing IL-6 secretion compared to both luteolin and ACHP treated THP-1 cells (Fig. 3B). Furthermore, this essential oil and linalool were able to reduce IL-6 protein level below the control level (Fig. 3B).

In case of IL-8 chemokine the two examined essential oils successfully downregulated its mRNA level, meanwhile the NFκB inhibitors did not changed significantly the IL-8 mRNA level compared to the LPS treatment (Fig. 3C). These results assume that linalool and LEOs may act other mode and/or not only via the NFκB pathway. Interestingly, at protein level only LEO/beginning of flowering period decreased IL-8 secretion, but it was still significantly higher level compared to control (Fig. 3D).

Again, LEO/beginning of flowering period was able to decrease to the highest extent the IL-1β mRNA level among the three essential oils and compared to both luteolin and ACHP treatments (Fig. 3E). However, both linalool and LEO/end of the flowering period were also significantly reduced the IL-1β mRNA level compared to the LPS treatment (Fig. 3E). Considering the IL-1β protein secretion, significant decrease was observed in case of LEO/beginning of flowering period as well as in case of luteolin and ACHP treated THP-1 cells, but not in case of the other two examined essential oils (Fig. 3F). This discrepancy between the mRNA and protein levels may be due to the post-translational maturation process of IL-1β.

Finally, the examination of TNFα mRNA synthesis revealed that only linalool could inhibit its expression, which effect was similar to the effect of NFκB inhibitors (Fig. 3G). Surprisingly, at protein level not only linalool, but LEO/beginning of flowering period were also effective on reducing TNFα secretion likewise luteolin and ACHP treatments (Fig. 3H).

Preventing function of pretreatments using linalool, LEOs and NFκB inhibitors on the mRNA expression and secretion of pro-inflammatory cytokines of THP-1 cells exposed to *P. aeruginosa* LPS.

The protective effect of linalool and LEOs on inflammation was also examined. Essential oil pretreatments were used for 24 h, which were followed by 24 h long LPS treatment. The effectiveness of the essential oils and linalool were compared to those of luteolin and ACHP treatments.

**Fig. 4:**
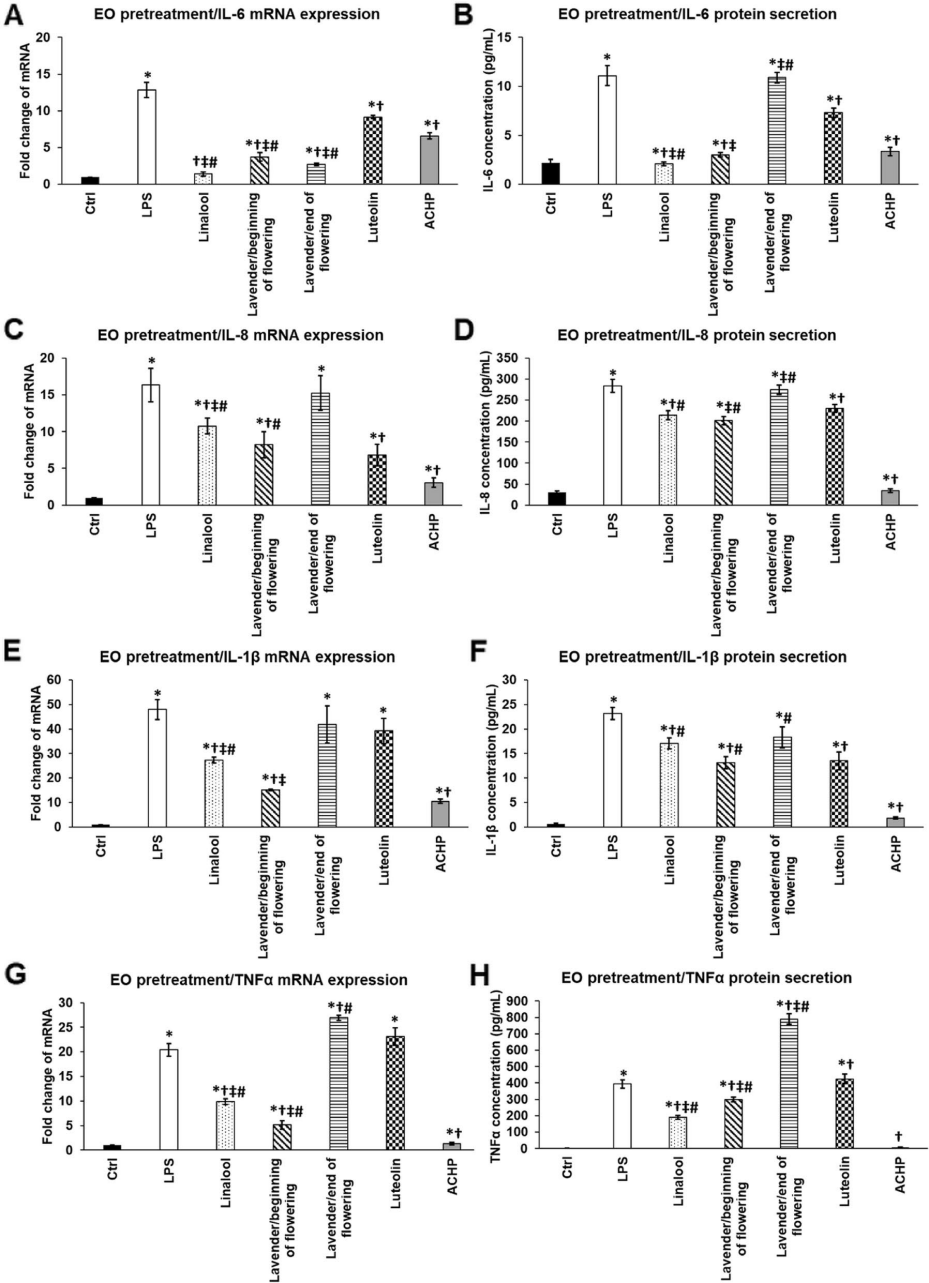
Effects of linalool and LEOs and NFκB inhibitors pretreatments on mRNA (**A,C,E,G**) and protein (**B,D,F,H**) levels of pro-inflammatory cytokines IL-6, IL-8, IL-1β and TNFα. THP-1 cells were pretreated with 500-fold diluted linalool, LEOs and 5 μM luteolin or ACHP for 24 h then 100 ng/mL *P. aeruginosa* LPS was added to the pretreated cells for 24 h. DMSO administration was used as a control of the treated cells. Real Time PCR was performed with SYBR green protocol using specific primers. β-actin was used as housekeeping gene for the normalization and the relative expression of controls was considered as 1. Pro-inflammatory cytokine secretions were determined using IL-6, IL-8, IL-1β and TNFα specific ELISA kits according to the manufacturer’s instructions. The bars represent mean values and error bars represent standard deviation (SD) for three independent determinations (*n* = 3). Real Time PCR and ELISA measurements were carried out in triplicate in each independent experiment. Asterisks indicate *p* < 0.05 compared to the control. Crosses show *p* < 0.05 compared to the LPS treatment. Double cross represents *p* < 0.05 compared to luteolin treatment. Number sign marks *p* < 0.05 compared to ACHP treatment. The diagrams were created using the same logical structure and the different treatments appeared in the same sequence in each figure in order to improve traceability of the results

In case of IL-6 linalool pretreatment was the most efficient in decresing mRNA expression, but it was also revealed that both LEOs were able to significantly decrease IL-6 mRNA level compared to both LPS treatment and to NFκB inhibitors (Fig. 4A). At protein level, both linalool and LEO/beginning of flowering period were successful in reducing IL-6 cytokine secretion and these levels were highly similar to the control level suggesting that these essential oils are potent inhibitors of IL-6 production (Fig. 4B).

LEO/beginning of flowering period showed the highest activity in attenuating the IL-8 mRNA expression among the three examined essential oils, although ACHP NFκB inhibitor was more effective in reducing IL-8 mRNA level than the essential oils (Fig. 4C). At protein level the same results were obtained (Fig. 4D).

Both IL-1β mRNA and protein levels were found to be altered similarly compared to IL-8. Linalool and LEO/beginning of flowering period were able to reduce IL-1β mRNA expression compared to LPS treatment, but neither LEO/end of flowering period nor luteolin were not auspicious to decrease it (Fig. 4E). At protein level LEO/beginning of flowering period was resultful effective in inhibiting IL-1β cytokine secretion, but this protein level was still significatly higher comparing to the secretion of the ACHP treated THP-1 cells (Fig. 4F).

In case of TNFα cytokine expression, linalool and LEO/beginning of flowering period were capable to inhibit mRNA synthesis, but the latter one was more effective (Fig. 4G). However, ACHP NFκB inhibitor was more successful in reducing TNFα mRNA level than LEO/beginning of flowering period (Fig. 4G). At protein level we found opposing results; linalool was better in decreasing TNFα secretion compared to LEO/beginning of flowering period, but still ACHP treatment was the most effective showing highly similar TNFα level as the control cells (Fig. 4H).

It is an interesting observation that LEO/end of flowering period showed as increased IL-6, IL-8 and IL-1β secretions as the LPS treated THP-1 cells. Moreover, pretreatment of THP-1 cells with LEO/end of flowering period resulted in two times higher TNFα secretion level compared to the LPS treated cells (Fig. 4H).

## Discussion

Lavender essential oil (LEO) is mainly used in aromatherapy, but it has several pharmacological and therapeutic properties [37]. LEO has a remarkable effect on anxiety, it can be used for relaxation by inhalation of the oil vapour [38]. It may be effective in treatments of several neurological disorders since it has neuroprotective features [39, 40]. Furthermore, it possesses anaesthetic activity [41] as well as antifungal [42] and antibacterial activities [43, 44]. The anti-inflammatory effect of LEO was tested in acute inflammation models of pleurisy and ear oedema [45, 46]. In mouse ear tissues it decreased the expression levels of NFκB, cyclooxygenase 2 and TNFα [47].

It has been described that LEO can increase phagocytic activity and modifies the intracellular bacterial proliferation in human macrophages by increasing reactive oxygen species production in case of *Staphylococcus aureus* infection [48].

The anti-inflammatory activity of essential oils may depend on the composition and the ratios of the compounds [36]. The composition of the essential oils extracted from the different stages of flowering period varies [49], which assumes that the collection time of the flowers influences the anti-inflammatory effects.

Alveolar macrophages, the cells of the innate immune system, are the major initial source of pro-inflammatory cytokines and chemokines including IL-1β, IL-6, IL-8 and TNF-α, which are secreted by the macrophages upon *P. aeruginosa* infection [50, 51]. These cytokines enhance the eradication of *P. aeruginosa,* but the high levels of these inflammatory molecules can cause severe damages to the lung tissue [52].

The aim of our study was to reveal if essential oils distilled from lavender flowers collected at the beginning and at the end of the flowering period could influence the pro-inflammatory cytokine production of *P. aeruginosa* LPS treated THP-1 human monocytes. LPS activates macrophage polarization to M1 state, which means that the macrophages exert pro-inflammatory activities, they release pro-inflammatory molecules e.g. nitric oxide, TNFα, IL-1β, IL-6 and prostaglandins, and they have increased phagocytic activity [53, 54]. The production of pro-inflammatory cytokines is mainly mediated by the NFκB signalling pathway [55].

According to literature, linalool and linalyl acetate possess anti-inflammatory effect by decreasing the phosphorylation of p65 and p50 NFκB transcription factors and consequently it reduces the production of pro-inflammatory cytokines IL-6 and TNFα [36, 56, 57]. Several essential oils (e.g. eucalyptus, tea tree or thyme) and their major components (eucalyptol, terpinene-4-ol and thymol) are able to reduce inflammation in different animal models and in in vitro cell cultures of microglia, monocytes and lymphocytes [36, 58–60].

The major components of lavender oil/beginning of flowering period we identified were 36.33% linalool and 21.47% linalyl acetate while of lavender oil/end of flowering period were 41.63% linalool and 14.78% linalyl acetate. Additional compounds with higher percentage were terpinene-4-ol (9.47 and 16.69%), α-terpineol (4.27 and 5.23%), geranyl acetate (3.19 and 2.92%), β-ocimene (3.43 and 1.33%), lavandulyl acetate (2.35 and 1.1%) and β-farnesene (1.51 and 1.3%). The presence of these compounds and additional ones even in very small amounts may modify the anti-inflammatory effects of the examined LEOs. It has been described that terpinene-4-ol can suppress the release of inflammatory mediators of macrophages [61], while α-terpineol decreases the production of IL-6 pro-inflammatory cytokine [62]. Both geranyl acetate and lavandulyl acetate may contribute to the anti-inflammatory properties of LEO, the latter one is able to reduce the production of IL-6 and IL-8 in keratinocytes [63]. β-ocimene is a potent molecule with anti-inflammatory activity [64] and β-farnesene possesses antimicrobial activity against *P. aeruginosa* [65]. Caryophyllene, caryophyllene oxide and α-humulene were also determined as minor compounds of both LEOs. These sesquiterpenes are known to be implicated in the anti-inflammatory activity of the essential oils by inhibiting the TLR signalling cascade and the activation of NFκB to release of inflammatory mediators by the immune cells [66].

To measure the effectiveness of LEOs on reducing inflammation, two selective NFκB inhibitors, which modulate the activity of the pathway at distinct levels, were used as positive controls. Luteolin inhibits the degradation of IκBα and nuclear translocation of NFκB p65 subunit and inhibits the LPS-induced TNFα, IL-6 production [31, 32] by decreasing the LPS-induced DNA binding activity of activating protein-1 [32] and IκB kinase (IKK) phosphorylation [67]. ACHP is an IKK inhibitor, which is selective for IKKα and IKKβ [33]. ACHP also blocks DNA binding activity of NFκB and STAT3 signalling [34].

Treatments of THP-1 cells with linalool and LEOs revealed that linalool and LEO/end of flowering period only increased IL-6 production suggesting that essential oils may influence NFκB pathway, but this effect was not significant. The increasing level of IL-6 may show the autocrine effect of the cytokine. IL-6 activates the JAK/STAT3 pathway via its receptor, which has a synergistic effect with the NFκB pathway and they mediate together the expression of IL-6 [68]. Moreover, the downregulation of IL-8 and IL-1β suggests that the essential oils alone did not activate the differentiation of monocytes.

IL-6 mRNA expression as well as secretion were decreased using linalool or LEO/beginning of flowering period even after LPS pretreatment or LPS treatment following essential oil pretreatments and their effect was stronger compared to the NFκB inhibitors. These results suggest that linalool and LEO/beginning of flowering period influence multiple regulatory points of the NFκB pathway and may also inhibit JAK/STAT3 signalling.

IL-8 is a major chemokine that stimulates the migration and the activity of monocytes and macrophages [69]. Its secretion is upregulated by LPS stimulation and it acts both autocrine and paracrine ways [70]. Moreover, IL-8 can modulate the production of IL-6 and IL-1β but not TNFα suggesting that although IL-8 triggers NFκB transcription factor activity, but does not affect TNFα promoter activity [71]. In our study linalool or LEO/beginning of flowering period were able to decrease IL-8 mRNA level and protein levels in case of LPS pretreatment. Moreover, LEO/beginning of flowering period was more successful in decreasing IL-8 level compared to the NFκB inhibitors. However, these promising results were less prominent in case of essential oil pretreatment. The aforementioned essential oils reduced IL-8 production, but ACHP NFκB inhibitor was more efficient compared to them suggesting that the NFκB inhibitor activity was lower in case of the essential oils.

Interestingly, IL-1β synthesis is mediated by TLR4 upon LPS stimulation but it is not enough for IL-1β secretion [72]. IL-1β mRNA transcription is activated by the NFκB signalling pathway. IL-1β is translated as a precursor molecule and it is converted to an active form by capsase-1. This enzyme is the part of the inflammasome, which is activated by TLR4 signalling [73]. It has been proven that the infection of macrophages with different bacteria strains e.g. *Mycobacterium tuberculosis* and *Pseudomonas aeruginosa* induces IL-1β secretion via the activation of an inflammasome [74, 75]. Moreover, *P. aeruginosa* triggers macrophage autophagy by increased IL-1β secretion and as a consequence IL-1β suppresses killing capacity of macrophages [50]. Therefore downregulation of IL-1β secretion of macrophages would be beneficial for increasing the activity of macrophages against *P. aeruginosa* infection. Interestingly, in case of LPS pretreatment it seems that both examined essential oils were able to decrease IL-1β mRNA expression, but only LEO/beginning of flowering period reduced the secretion of IL-1 β compared to the LPS treatment. These observations propose that the essential oils may inhibit NFκB pathway, but cannot block the activation of the inflammasome and therefore the secretion of IL-1β. The possible reason for the action of the essential oils is that *the inhibition of IKKβ, an activator of* NFκB *can induce IL-1β secretion following LPS treatment* [76]*.* Linalool and LEO/beginning of flowering period showed more promising results in case of pretreatments of THP-1 cells. Both of them effectively downregulated the mRNA and protein levels of IL-1β pro-inflammatory cytokine suggesting that they may prevent the activation of both NFκB and inflammasome.

It seems that the examined essential oils were less effective on reducing TNFα expression. The reason for this diversion could be explained by the mechanism of action of TNFα. TNFα stimulates the proliferation of the macrophages as well as the differentiation towards the M1 state [77]. Therefore, it has a pivotal role in the regulation of the inflammatory cytokine production by activating the NFκB pathway via the degradation of IκBα and the activation of the NFκB subunits [78]. Moreover, the autocrine action of TNFα mediated by the TLR4 activation contributes to a long-term survival of macrophages [79]. After LPS activation only linalool was able to significantly decrease TNFα mRNA expression, meanwhile TNFα secretion was significantly lower in case of both linalool and lavender oil/beginning of flowering period treatments. Furthermore, these levels were similar to those in case of luteolin and ACHP treatments. Pretreatment with linalool and lavender oil/beginning of flowering period were successful in decreasing TNFα, but ACHP was significantly better compared to the essential oils. The possible explanation for these results is that LPS activates the canonical pathway and the release of the heterodimer p65/p50 as well as the alternative/non-canonical pathway comprising the release of the heterodimer RelB/p52. The latter one inhibits TNFα production in macrophages [80]. Linalool and LEO/ beginning of flowering period may influence both NFκB pathways, which have opposite effects on TNFα expression.

Although LEO/end of flowering period decreased IL-6, IL-1β and IL-8 mRNA expression in case of LPS pretreatment, it was not capable to reduce cytokine secretion. Furthermore, LEO/end of flowering period mainly increased pro-inflammatory cytokine production rather than inhibit it in case of essential oil pretreatment.

The synergism between the components of the examined essential oils may contribute to their anti-inflammatory properties. The differences in the composition of lavender oil extracted at the beginning and at the end of flowering period may participate in their distinct effects on the regulation and production of IL-6, IL-1β, IL-8 and TNFα pro-inflammatory cytokines in *P. aeruginosa* LPS-stimulated THP-1 cells.

## Conclusions

In our study, we revealed the different anti-inflammatory effects of LEOs extracted from both the beginning and at the end of flowering period on THP-1 human monocyte/macrophage cell line. In our experiments *P. aeruginosa* LPS treatment was used for inducing inflammation according to its high relevance in respiratory infections throughout the world. We determined the mRNA as well as the protein concentrations of potent pro-inflammatory cytokines IL-6, IL-8, IL-β and TNFα produced by macrophages at inflammation. To prove the effectiveness of the LEOs and their major component linalool, we utilised well-known inhibitors of pro-inflammatory cytokine synthesis. According to our results, it seems that the differences in the composition of the examined LEOs contribute to the anti-inflammatory effects. Based on our results it has been proven that LEO extracted at the beginning of flowering period is a potent inhibitor of the synthesis of four pro-inflammatory cytokines IL-6, IL-8, IL-β and TNFα of THP-1 cells. This supports the relevance of the collection of the lavender flowers form early blooming period for essential oil production and for the utilization as an anti-inflammatory treatment.

## Supplementary Information

**Additional file 1.**

## Data Availability

All data generated or analysed during this study are included in this published article.

## Abbreviations

ACHP: 2-Amino-6-[2-(cyclopropylmethoxy)-6-hydroxyphenyl]-4-(4-piperidinyl)-3-pyridinecarbonitrile
AP-1: Activator protein-1
CCK-8: Cell counting kit-8
DMSO: Dimethyl sulphoxide
ELISA: Enzyme-linked immunosorbent assay
EMA: European medicines agency
FBS: Fetal bovine serum
GC-FID: Gas chromatography flame ionisation detector
GC-MS: Gas chromatography mass spectrometer
IKK: IκB kinase
IL-1β: Interleukin-1β
IL-6: Interleukin-6
IL-8: Interleukin-8
IRF3: Interferon regulatory factor 3
JAK: Janus kinase
LEO: Lavender essential oil
LPS: Lipopolysaccharide
LRI: Linear retention index
MAPK: Mitogen activated protein kinase
NFκB: Nuclear factor kappaB
PBS: Phosphate buffered saline
STAT3: Signal transducer and activator of transcription 3
TLR4: Toll-like receptor 4
TNFα: Tumor necrosis factor α
