# Supplementary material for: Anti-inflammatory effect of lavender (Lavandula angustifolia Mill.) essential oil prepared during different plant phenophases on THP-1 macrophages
Source: BMC Complement Med Ther. 2021 Nov 24;21:287. doi: 10.1186/s12906-021-03461-5 (PMC8611982; doi:10.1186/s12906-021-03461-5)
Supplement: Supplementary file 1 — Additional file 1. [file 12906_2021_3461_MOESM1_ESM.pdf]

Anti-inflammatory effect of lavender (Lavandula angustifolia L.) essential oil prepared during different plant phenophases on THP-1 macrophages

Edina Pandur [1], Alex Balatinácz [2], Giuseppe Micalizzi[3], Luigi Mondello[3,4,5], Adrienn Horváth [2], Katalin Sipos [1] and Györgyi Horváth [2,*]

[1] Department of Pharmaceutical Biology, Faculty of Pharmacy, University of Pécs, H-7624, Rókus u. 2., Pécs, Hungary; katalin.sipos@aok.pte.hu, edina.pandur@aok.pte.hu
[2] Department of Pharmacognosy, Faculty of Pharmacy, University of Pécs, H-7624, Rókus u. 2., Pécs, Hungary; horvath.gyorgyi@gytk.pte.hu, horvath.adrienn2@pte.hu, balatinacz.alex@gmail.com
[3] Chromaleont s.r.l., c/o Department of Chemical, Biological, Pharmaceutical and Environmental Sciences, University of Messina, 98168, Italy; giuseppe.micalizzi@chromaleont.it, lmondello@unime.it
[4] Department of Chemical, Biological, Pharmaceutical and Environmental Sciences, University of Messina, 98168, Italy; lmondello@unime.it
[5] Unit of Food Science and Nutrition, Department of Medicine, University Campus Bio-Medico of Rome, 00128 Rome, Italy; lmondello@unime.it

* Correspondence: horvath.gyorgyi@gytk.pte.hu

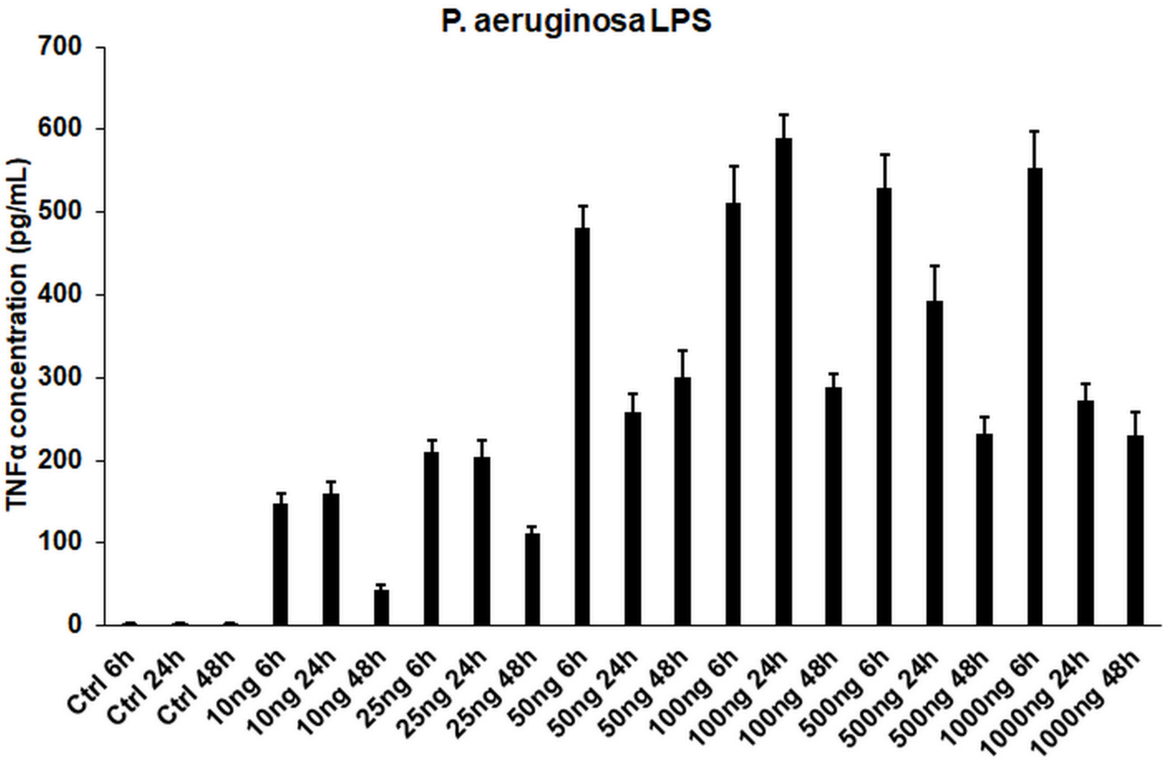

Supplementary Figure 1. Time and concentration dependence of TNFα secretion of THP-1 cells after *P. aeruginosa* LPS treatments. THP-1 cells were cultured in 6-well plates and were treated with 10, 25, 50, 100, 500 and 1000 ng/mL of P. aeruginosa LPS for 6 h, 24 h and 48 h. After the treatments, the cells were pelleted and the cell culture supernatant of each sample was used to determine the TNFα pro-inflammatory cytokine secretion using human TNFα specific ELISA kit. The columns show mean ± SD for three independent experiments. The treatments were carried out in triplicates in each independent experiment.

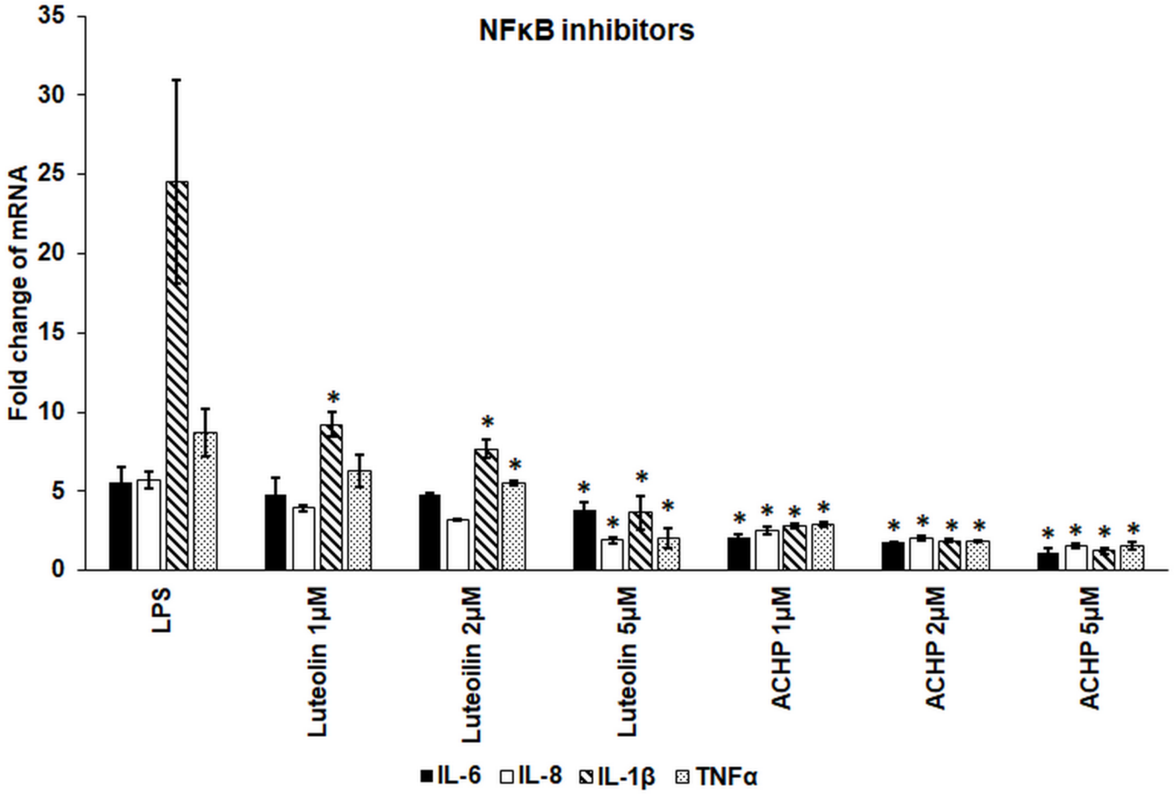

Supplementary Figure 2. The effects of NFκB inhibitors luteolin and ACHP on the pro-inflammatory cytokine mRNA expression of THP-1 cells. The THP-1 cells were preincubated with 1, 2 or 5 µM luteolin or ACHP for 30 min then the cells were treated with 100 ng/mL *P. aeruginosa* LPS for 24 h. After the treatments the cells were collected and total RNA was isolated from the samples. Complementary DNA form each sample was synthetized using 200 ng of total RNA. The mRNA expression levels of IL-6, IL-8, IL-1β and TNFα pro-inflammatory cytokines were determined with Real Time PCR using SYBR Green protocol. β-actin was used for normalization and the fold change of mRNA expression was calculated with Livak ($2^{-\Delta\Delta Ct}$) method. The columns represent mean of fold change ± SD for three independent experiments. The measurements were carried out in triplicates in each independent experiment. Asterisks indicate $p < 0.05$ compared to the LPS treated cells.
